# Plant chloroplast stress response: insights from mass spectrometry metabolites analysis

**DOI:** 10.3389/fpls.2025.1549156

**Published:** 2025-03-19

**Authors:** Si Cheng, Jiawei Xu, Siqi Wu, Qun Li, Jianing Mi

**Affiliations:** ^1^ Chinese Medicine Guangdong Laboratory, Guangdong-Macao In-Depth Cooperation Zone in Hengqin, Zhuhai, China; ^2^ State Key Laboratory of Traditional Chinese Medicine Syndrome, The Second Affiliated Hospital of Guangzhou University of Chinese Medicine (Guangdong Provincial Hospital of Chinese Medicine), Guangzhou, China

**Keywords:** chloroplast, mass spectrometry, metabolite analysis, oxidative stress, reactive oxygen species, retrograde signals, stress response

## Abstract

Plant chloroplasts produce excess reactive oxygen species (ROS) during photosynthesis, particularly under biotic and abiotic stress conditions. These adverse environmental stresses lead to significant alterations in various cellular components, especially within the chloroplast, which serves as a key stress-sensor organelle. The stress response of chloroplasts can trigger plastid-to-nucleus retrograde signaling and enhance the biosynthesis of biologically active compounds and phytohormones, which are mechanisms that aid plants in acclimating to environmental stress. While ROS act as signaling molecules to help re-adjust cellular metabolic homeostasis, they also risk damaging chloroplasts’ structural and functional integrity. Recent research on stress-induced plant metabolism has provided new insights into the chloroplast’s stress response. In particular, advancements in mass spectrometry (MS) techniques have expanded our understanding of how oxidative stress affects plants through metabolomics analyses of metabolites involved in this process. Here, we emphasize the MS-based profiling of lipids, apocarotenoids, and phytohormones linked to ROS-triggered processes in plants. Moreover, we discuss the plants’ metabolic responses to abiotic stress. Finally, we outline future directions for chloroplast stress research. We advocate for integrating MS-based metabolomics with biochemical and molecular genetic approaches to discover new signaling molecules and identify interconnected signaling components that function across multiple chloroplast signaling pathways.

## 1 Introduction

The chloroplast is an organelle that hosts numerous essential metabolic pathways, including photosynthesis, and facilitates the conversion of radiant energy into chemical energy (Heinig et al., 2013; Zoschke and Bock, 2018). Additionally, chloroplasts are crucial in monitoring plant surroundings and signaling for acclimation (Woodson, 2019). Adverse abiotic and biotic stresses disrupt cellular homeostasis and trigger chloroplast stress responses. This leads to the formation of plastid-to-nucleus retrograde signaling (RS) and enhances the biosynthesis of biologically active compounds and phytohormones, which mediate plant acclimation to environmental stresses (Chan et al., 2016; Noctor et al., 2018; Woodson, 2019). Upon sensing adverse ecological conditions, chloroplasts inevitably produce harmful by-products, such as ROS (Noctor et al., 2018; Shapiguzov et al., 2020). While ROS can significantly damage chloroplasts’ structural and functional integrity (Halliwell, 2006; Shapiguzov et al., 2020), they also perform essential signaling functions by helping to readjust cellular metabolic homeostasis and facilitating cell repair (Halliwell, 2006; Foyer and Noctor, 2016; Foyer et al., 2017; Sies, 2017; Woodson, 2019; Kim, 2020). In addition to ROS, the chloroplast stress response modulates the biosynthesis of secondary metabolites, including signaling molecules like β-cyclocitral (β-CC) and precursors of stress hormones such as 12-oxo-phytodienoic acid (12-OPDA) and xanthoxin (Considine and Foyer, 2021). Compelling evidence indicates that chloroplast stress-associated metabolites, such as β-CC and 3′(2′)-phosphoadenosine-5′-phosphate (PAP), play pivotal roles in maintaining optimal chloroplast functions in response to environmental changes in plants (Wagner et al., 2004; Møller et al., 2007; Pogson et al., 2008; Dizengremel et al., 2009; Ramel et al., 2012; Ramel et al., 2013; Kleine and Leister, 2016; Fichman et al., 2020; Li and Kim, 2021; Moreno et al., 2021b). With advancements in MS, large-scale analyses can identify and quantify numerous plant metabolites through metabolomics approaches (Fernie et al., 2004; Sato et al., 2004; Mi et al., 2016b; Zhang et al., 2017). Furthermore, elucidating the metabolism of chloroplast signaling-associated molecules under various stress conditions may provide essential insights into the impact of chloroplast stress signaling on cellular homeostasis and plant resilience (Mi et al., 2018b; Mi et al., 2019b; Moreno et al., 2021a; Jia et al., 2022; Mi et al., 2022a; Mi et al., 2022b). In this work, we focus on key metabolites involved in the chloroplast stress response, provide an overview of MS analysis of these metabolites, and discuss future applications of MS research.

## 2 The alteration of plant metabolism in chloroplast stress responses

Environmental stresses can adversely affect various cellular components, including nucleic acids, proteins, and lipids, by altering their structure and function. As a stress sensor, the chloroplast is particularly vulnerable to these adverse environmental conditions, which can lead to changes in the redox state of proteins, gene expression, and the production of ROS. These changes can impact plant metabolism at multiple levels through diverse mechanisms (Noctor et al., 2015). For instance, there is a direct increase in the production of oxidized compounds due to ROS-triggered chemical reactions. A notable example of this is the oxidative cleavage of carotenoids, leading to the formation of apocarotenoids (Ramel et al., 2012; Ramel et al., 2013). ROS production can also influence enzyme activity, indirectly affecting plant metabolism (Zaffagnini et al., 2012; Li and Kim, 2021). Oxidative stress, for instance, can modify the TCA cycle by impacting ascorbate metabolism in plants (Millar et al., 2003; Nunes-Nesi et al., 2005; Ishikawa et al., 2010; Zaffagnini et al., 2012; Dumont and Rivoal, 2019; Savchenko and Tikhonov, 2021). Changes in the thiol redox state within chloroplasts under oxidative stress may also affect plant metabolism, including glutathione metabolism (Sies, 2017; Dorion et al., 2021). Moreover, ROS-dependent metabolic adjustments can lead to secondary effects, as altered metabolite levels can further activate signaling, defense, or acclimatory pathways (Noctor et al., 2015; Li and Kim, 2021). For example, producing nicotinamide adenine dinucleotide phosphate (NADPH) is crucial for maintaining intracellular detoxification in plants. Adequate levels of NADPH result from increased activity in various catabolic pathways and associated shunts, such as glucose-6-phosphate dehydrogenase, NADP-dependent glyceraldehyde-3-phosphate dehydrogenase, and isocitrate dehydrogenase (Dizengremel et al., 2009). Thus, alterations in the activity of any of these enzymes could disrupt NADPH levels, ultimately affecting the plant’s sensitivity or tolerance to oxidative stress conditions.

## 3 MS strategies for analyzing chloroplast stress response-derived metabolites

Various MS-based platforms with good sensitivity and a wide dynamic range have been utilized in the comprehensive profile of chloroplast stress response-derived metabolites from a complex biological plant sample, such as gas chromatography-mass spectrometry (GC–MS) and liquid chromatography-mass spectrometry (LC–MS) (**Table 1**).

**Table 1:** MS analyses of oxidative stress markers in chloroplast stress responses.

Markers	Stresses	Biological function	MS techniques
Lipid peroxides, e.g., MDA, HNE	Thermal stress	Stimulating gene expression and cell survival; Inhibiting gene expression and promoting cell death	GC-MS LC-MS
Oxylipins	Wounding; Insect attacks; Pathogen	Signaling molecules for defense and development	LC-MS
Galactolipid, Triacylglyceride	Cold; Drought	Storage lipids; Lipids of the plasma membrane	LC-MS
Zeaxanthin, Antheraxanthin	High light	Regulating xanthophyll cycle-based photoprotective system	LC-MS
Apocarotenoids, e.g., β-CC, β-CCA, DHA	High light; Drought	Signaling molecules; Root growth promoter; Precursors of ABA	GC-MS LC-MS
GAs	Salinity	Plant hormone	GC-MS LC-MS
ABA	Drought; Salinity	Plant hormone	LC-MS
Auxins	Drought; Salinity	Plant hormone	GC-MS
JA	Wounding	Plant hormone	GC-MS LC-MS

GC-MS technology is a highly effective analytical technique for analyzing samples in complex matrices. It offers excellent separation and resolution of metabolites, enabling the detection and quantification of known and unknown metabolites, even at low concentrations, with remarkable reproducibility (Misra, 2021). GC-MS strategies are adept at achieving extensive coverage of diverse compound classes, encompassing polar organic compounds, amino acids, hydrophilic carbohydrates, and hydrophobic lipids, particularly after suitable derivatization steps. This analytical technique excels in separating and quantifying metabolites with high sensitivity and reproducibility. However, it is essential to note that GC-MS technology is constrained in its ability to analyze volatile and thermally stable metabolites and compounds that lack the potential for chemical modification to yield volatile derivatives (Fiehn, 2016). The electron ionization (EI) method is predominantly utilized in GC-MS to ensure high reproducibility in analytical results. The most common mass analyzers employed in GC-MS are Quadrupole (Q) and orthogonal time-of-flight (oTOF) analyzers. Q-MS analyzers offer exceptional sensitivity for targeted compound analysis and boast a broad dynamic range. In contrast, TOF-MS analyzers deliver superior mass accuracy, enhanced duty cycles, and accelerated acquisition times, making them a valuable option for comprehensive metabolomic studies.

LC-MS is a pivotal technology for analyzing thermolabile and polar metabolites and high-molecular-weight compounds without derivatization. The versatility of column chemistry and retention mechanisms in LC-MS allows for broad coverage of the plant metabolome (Marta et al., 2021). For instance, reversed-phase stationary phases facilitate strong interactions with less-polar compounds, while normal-phase stationary phases are employed to separate highly polar metabolites effectively. However, hydrophilic interaction liquid chromatography (HILIC) and porous graphitic carbon stationary phases are gaining prominence for analyzing highly polar metabolites commonly encountered within the plant metabolome. Electrospray ionization (ESI) is LC-MS analyses’ most widely used ionization method. In addition to triple quadrupole (QQQ) MS and time-of-flight (TOF) MS, Orbitrap-MS represents a high-resolution mass spectrometry system increasingly employed for qualitatively analyzing plant metabolites.

## 4 MS analysis of lipids and their peroxides

Chloroplast oxidative stress induces lipid peroxidation (LPO), a complex process in which free radicals attack polyunsaturated fatty acids, synthesizing lipid peroxides (Vollenweider et al., 2000; Farmer et al., 2003; Davoine et al., 2006). The lipid peroxides encompass aldehydes, ketones, alcohols, ethers, and alkanes (Noctor et al., 2015). LPO can either result in plant oxidative injury or promote plant redox homeostasis (Noctor et al., 2015). LPO can occur through both enzymatic and non-enzymatic processes. In the enzymatic process, chloroplastic lipoxygenases serve as an early step in the synthesis of jasmonates (JAs) (Bell and Mullet, 1993; Farmer et al., 2003), and play a crucial role as they catalyze the peroxidation of fatty acids, particularly during wounding stress. Additionally, lipoxygenases catalyze the oxidation of polyunsaturated fatty acids, producing bioactive oxylipins that are activated in response to various stress conditions, including wounding and insect and pathogen attacks (Feussner et al., 1995; Göbel et al., 2001). In non-enzymatic reactions, ROS are the primary agents triggering the generation of lipid peroxides (Oenel et al., 2017; Mhamdi and Van Breusegem, 2018). Some of these lipid peroxides have been studied as markers of oxidative stress (e.g., malondialdehyde [MDA]), as well as lipid-derived signaling molecules and hormones (e.g., JAs), particularly under stress conditions (Feussner et al., 1995; Göbel et al., 2001; Biswas and Mano, 2015; Choudhury et al., 2017; Oenel et al., 2017; Yonny et al., 2017; Mhamdi and Van Breusegem, 2018; Alché, 2019).

MS-based lipid profiling has been utilized to investigate the effects of stress on lipid metabolism and the oxidation mechanisms associated with stress-induced LPO in plants. For instance, Yonny et al. developed an ultra-high-performance liquid chromatography (UHPLC)-MS method for detecting the oxidative stress marker MDA directly from the leaves of melon plants under thermal stress conditions, eliminating the need for derivatization (Yonny et al., 2017). Additionally, another significant lipid peroxide, 4-hydroxy-2-nonenal (HNE), a reactive aldehyde, was measured in carrot callus cultures using an LC-MS approach, which aids in studying free radical-mediated mechanisms during *in vitro* plant development (Deighton et al., 1997). Beyond analytical protocols for detecting free-form aldehydes, high-sensitivity LC-MS techniques have been developed utilizing derivatization to detect various aldehydes from plant samples (Deighton et al., 1997; Yonny et al., 2017; Jia et al., 2019; Mi et al., 2019a; Mi et al., 2020; Jia et al., 2021). These LC-MS approaches will be critical tools for analyzing lipid peroxides in chloroplast oxidative stress-induced LPO.

In addition, MS-based metabolomics analytical strategies, particularly lipidomics, have been extensively employed to investigate alterations in lipid metabolism in plants under stress conditions. Using an ultra-performance liquid chromatography (UPLC)-MS method (Hummel et al., 2011), Degenkolbe et al. conducted a comparative analysis of 180 lipid species across 15 Arabidopsis accessions, revealing significant accumulation of storage lipids, particularly long-chain unsaturated triacylglycerides, in most accessions following cold acclimation (Degenkolbe et al., 2012). Introducing ion trap (IT) technology into the analytical workflow has enhanced high-resolution MS^n^, making it an efficient method for characterizing complex mixtures of plant metabolites (Okazaki et al., 2013; Higashi et al., 2015). Higashi et al. applied HILIC coupled with IT-time-of-flight MS for lipidomic analysis, detecting 66 glycerolipid species in Arabidopsis under heat stress (Wang et al., 2014). Their results indicated that certain glycerolipid species increased while others decreased during recovery from stress exposure, suggesting a reduction in unsaturated fatty acids from chloroplast membranes (Higashi et al., 2015). Furthermore, high-sensitivity triple quadrupole-tandem MS (QQQ-MS/MS) has also been utilized in targeted lipidomic experiments (Wang et al., 2014; Mi et al., 2016a; Mi et al., 2018a). For instance, Tarazona et al. examined lipid alterations related to cold and drought stress using UPLC-QQQ-MS/MS, revealing close relationships between glycosyl inositol phosphor ceramides, steryl glycosides, and acylated steryl glycosides in response to drought stress (Tarazona et al., 2015). Additionally, Zoeller et al. conducted targeted lipidomic analyses of LPO in the interaction between Arabidopsis wild-type and lipoxygenase 2 (*lox2*) mutants, which are deficient in 9-lipoxygenases and 13-lipoxygenases, and Pseudomonas syringae using UPLC-QQQ-MS/MS (Zoeller et al., 2012). Their findings indicated that LPO predominantly targeted plastid lipids, including galactolipid and triacylglyceride species (Zoeller et al., 2012).

## 5 MS analysis of carotenoids and their oxidation cleavage products

Many compounds, including carotenoids, have been reported as effective antioxidants capable of scavenging ROS in cells (Michaeli and Feitelson, 1994; Bouvier et al., 2005; Krieger-Liszkay and Trebst, 2006; Ramel et al., 2012). Their oxidation products and derivatives are recognized as stable and promising markers in plant oxidative stress responses (Ramel et al., 2012). Due to their roles as ROS scavengers, the antioxidant functions of carotenoids have been extensively studied in both humans and plants (Rao and Rao, 2007; Cazzonelli and Pogson, 2010; Avendaño-Vázquez et al., 2014; Fiedor and Burda, 2014; Swapnil et al., 2021). Although it remains unclear whether carotenoids are primary contributors to ROS homeostasis in plants, particularly under stress conditions, they are considered critical in regulating the accumulation of ROS and their derived molecules within the membrane phase (Swapnil et al., 2021). Additionally, carotenoids serve accessory light-harvesting roles and possess photoprotective properties, such as quenching triplet chlorophyll and singlet oxygen (^1^O_2_) through the xanthophyll cycle (Swapnil et al., 2021). Interestingly, stress conditions like high light exposure can stimulate the production of zeaxanthin and antheraxanthin, particularly influencing the ratio between these two compounds (Logan et al., 1998). Beyond their biophysical interactions, carotenoids can be oxidized to yield apocarotenoids, a reaction that can occur spontaneously through ROS or be catalyzed by enzymes from the CAROTENOID CLEAVAGE DIOXYGENASE (CCD) family (Havaux, 2014; Jia et al., 2018; Mi and Al-Babili, 2019). CCDs comprise a superfamily of mononuclear non-heme iron proteins that catalyze the oxygenolytic fission of alkene bonds in carotenoids to generate apocarotenoid products (Havaux, 2014; Jia et al., 2018; Mi and Al-Babili, 2019). Recent studies have identified various apocarotenoids associated with photoacclimation and drought tolerance, such as cyclic and acyclic apocarotenoids that confer high light and drought tolerance, including β-CC, β-cyclogeranic acid (also known as β-cyclocitric acid, β-CCA), and dihydroactinidiolide (DHA) (Moreno et al., 2021b).

(U)HPLC-UV approaches are preferred for detecting plant carotenoids due to their high abundance and characteristic UV absorption (Gupta et al., 2015). However, MS is employed to identify or detect carotenoids in organisms or materials where carotenoid levels are significantly low (Rivera et al., 2011; Pérez-Gálvez and Roca, 2018). While plants typically contain high levels of carotenoids, profiling of apocarotenoids is often conducted using GC or LC analytical platforms. For example, using GC-MS, Ramel et al. investigated the effects of light stress on β-carotene oxidation in Arabidopsis. Their results showed a rapid accumulation of β-carotene oxidation products, such as β-CC, β-ionone, and DHA, in plants exposed to high light stress within hours (Ramel et al., 2012). In conjunction with transcriptomics analysis, they concluded that β-CC acts as a signaling molecule produced during photooxidative stress and likely functions in the ^1^O_2_ signaling pathway in Arabidopsis (Ramel et al., 2012). In another study, Alexandra et al. used a UHPLC-MS method to detect β-CC in Arabidopsis roots, finding that β-CC enhances root branching in the presence of N-(4-fluoro-benzyl)-N-hydroxy-3-(4-methoxy-phenyl)-propionamide (D15). This CCD inhibitor reduces primary root length and inhibits lateral root capacity by 50%. This suggests that β-CC is a growth promoter in Arabidopsis roots (Alexandra et al., 2019). Recently, d’Alessandro et al. identified a water-soluble β-CCA in Arabidopsis leaves from plants subjected to drought stress or treated with exogenous β-CC using GC-MS (d’Alessandro et al., 2019). They also demonstrated the positive effects of exogenous β-CCA application on plant adaptation to drought stress, a benefit observed in other plants such as pansy, pepper, and tomato (d’Alessandro et al., 2019). Transcriptomic analysis of Arabidopsis plants exposed to excessive light or β-CC revealed that both treatments induce the expression of several glycosyltransferase genes in leaves (Ramel et al., 2012). Our group recently identified a new modification of apocarotenoids glycosylation resulting in the formation of apocarotenoid glucosides in Arabidopsis through MS-based metabolite profiling (Mi et al., 2019b). Furthermore, the results indicated that high-light conditions increase the levels of glycosylated apocarotenoids, including glycosylated β-CC, glycosylated β-ionone, glycosylated β-apo-11-carotenal, glycosylated β-apo-13-carotenone, and their isomers in Arabidopsis (Mi et al., 2019b). Additionally, we developed a UHPLC-MS method for apocarotenoid profiling. We used it to investigate the effect of salt stress on the metabolism of apocarotenoids in Arabidopsis, revealing that salt stress significantly induces the formation of C15 β-apo-11-carotenals, which are precursors of abscisic acid (ABA) in an alternative ABA1-independent biosynthetic pathway in plants (Jia et al., 2022).

## 6 MS analysis of chloroplast stress-related phytohormones

Chloroplasts are the primary sites for synthesizing phytohormone precursors, which are crucial in producing essential hormones such as gibberellic acid (GA), ABA, auxins, and JA. These phytohormones regulate various aspects of plant growth and development and mediate responses to environmental stimuli. Specifically, GA is instrumental in promoting seed germination and flowering, while ABA is vital for managing stress responses, such as stomatal closure during drought conditions (Peng and Harberd, 2002; Lim et al., 2015). Auxins facilitate cell elongation and are key to maintaining apical dominance and supporting root development (Gomes and Scortecci, 2021). Additionally, JA is critical for activating plant defense mechanisms against biotic and abiotic stressors (Wang et al., 2020). The biochemical processes occurring within chloroplasts significantly contribute to the overall balance of phytohormones, which is essential for the physiological functions of plants.

Increasing evidence suggests that phytohormones mediate oxidative stress signaling in plants (Zhu, 2002; Achard et al., 2006; Achard et al., 2008; Magome et al., 2008; Tognetti et al., 2010; Colebrook et al., 2014; Gao et al., 2014; Ruiz-Sola et al., 2014; Zhu, 2016; Sato et al., 2018; Wang et al., 2018; Balfagón et al., 2019). Phytohormone signaling allows for flexible and appropriate modulation of plant growth in response to environmental changes. For instance, Achard et al. detected bioactive GA1 and GA4 in 14-day-old wild-type Arabidopsis seedlings grown under 100 mM NaCl and control conditions using GC-MS (Achard et al., 2006). Their results showed that salt stress significantly reduced the levels of bioactive GAs. Studies of quadruple-DELLA and GA-deficient mutants concluded that salt-activated signaling pathways enhance the growth-repressing effects of DELLAs, at least partly by reducing bioactive GA levels (Achard et al., 2008). Similarly, Magome et al. profiled 15 GAs, including 13-H GAs and 13-OH GAs, from Arabidopsis wild-type and ga2ox7-2 mutant lines under high-salinity stress using LC-MS. They demonstrated that endogenous GA levels were actively reduced due to the induction of GA 2-oxidase regulated by the salinity-responsive DWARF AND DELAYED FLOWERING 1 (*DDF1*) gene, leading to the conclusion that growth is repressed to favor stress adaptation (Magome et al., 2008).

The role of ABA in abiotic stress responses is well-documented, with its signaling pathway central to drought and salt stress responses in plants (Zhu, 2002). Sato et al. measured ABA content in Arabidopsis wild-type and NGATHA1 (NGA1, a transcriptional regulator of NINE-CIS-EPOXYCAROTENOID DIOXYGENASE 3 [NCED3]) transgenic lines, both with and without dehydration stress, using LC-MS/MS (Sato et al., 2018). Their results indicated that dehydration stress enhances the NGATHA1 transcription factor, positively regulating ABA accumulation by activating the *NCED3* gene, a key player in ABA biosynthesis during early drought stress (Sato et al., 2018). In another study, Ruiz-Sola et al. reported that salt stress can rapidly activate the carotenoid pathway, specifically in roots, likely leading to the production of ABA precursors necessary for sustained ABA production (Ruiz-Sola et al., 2014).

Recent research on catalase-deficient plants has uncovered meaningful interactions with auxins. For example, using GC-MS, Gao et al. compared auxin levels between Arabidopsis wild-type and *catalase 2-1* (*cat2-1*) mutant leaves (Gao et al., 2014). Their results indicated that *cat2-1* mutant leaves accumulate high levels of H_2_O_2_ under photorespiratory conditions, leading to decreased auxin levels. Conversely, *cat2-1* mutant leaves exhibited lower H_2_O_2_ content and elevated auxin levels under low light intensities (Gao et al., 2014). Additionally, microLC-MS/MS was employed to detect auxins and auxin derivatives in Arabidopsis wild-type and transgenic plants ectopically expressing UGT74E2, a UDP-glycosyl transferase induced in catalase-deficient plants (Tognetti et al., 2010). The results showed that these transgenic plants had increased concentrations of indole-3-butyric acid glucose ester (IBA-Glc), as well as higher levels of free indole-3-butyric acid (IBA) and conjugated indole-3-acetic acid (IAA). The altered IBA and IAA homeostasis significantly improved survival during drought and salt stress treatments (Tognetti et al., 2010).

As discussed earlier, intracellular oxidative stress can also activate pathways associated with JA. A study utilizing a reversed-phase C_18_ column UPLC coupled with QQQ-MS profiled JA, JA-Ile, ABA, salicylic acid (SA), and 12-OPDA in Arabidopsis plants subjected to a combination of high light and heat stress (Balfagón et al., 2019). Balfagón et al. revealed that JA is crucial for regulating several transcriptional responses specific to these stress conditions and proposed that JA may function as a signaling molecule regulating plant photosynthesis under high light and heat stress (Balfagón et al., 2019).

With the development of LC-MS-based targeted plant hormonomics, Šimura et al. performed comprehensive profiling of 101 phytohormones using fresh plant material (Šimura et al., 2018). They highlighted key hormone metabolites, such as GA, ABA, auxins, and cytokinins, that play critical roles in plant adaptation to salt stress (Šimura et al., 2018). Additionally, Mi et al. utilized the targeted plant hormonomics strategy developed by Šimura et al. to investigate hormone levels regulated by manipulating carotenoid metabolism in crops, ultimately enhancing tomato’s abiotic stress tolerance to high light, salt, and drought conditions (Mi et al., 2022b).

## 7 Perspective

Our understanding of how chloroplast stress regulates plant metabolism and its positive contributions to plant adaptation to environmental stress has dramatically advanced in recent years. The impact of environmental stress on plant metabolism is highly complex. However, the widespread application of MS techniques in analyzing stress-regulated metabolites has enabled a more comprehensive understanding of metabolic regulation under stress conditions. Lipid oxidation enhances the metabolic flux of JA and signaling lipid peroxides in plants, which may serve as a signaling mechanism for environmental stress responses. Furthermore, the non-enzymatic oxidation of carotenoids not only scavenges ROS in the chloroplast but also likely generates important signaling apocarotenoids, including β-CC and precursors of ABA.

Environmental stresses stimulate hormone metabolism and signaling pathways, which regulate plants’ stress responses. However, new RS metabolites, such as novel apocarotenoids and their metabolic and signaling pathways, remain elusive. To address these biological questions, new multi-omics strategies should aid in identifying and characterizing the genes, enzymes, and metabolites involved. β-CC has been established as a new RS and plant growth regulator. However, information about its tissue-specific functions, transport, and plant perception remains largely unknown. In addition to β-CC, many carotenoid derivatives of unknown identity (e.g., linear cis-carotene-derived apocarotenoids, LCDAs) have been reported to possess signaling functions in plant development and environmental interactions (Escobar-Tovar et al., 2021; Moreno et al., 2021b; Dhami et al., 2022; Sierra et al., 2022). Recently identified C15 β-apo-11-carotenals can be induced by salt stress, contributing to enhanced ABA content in Arabidopsis (Jia et al., 2022). However, information regarding the genes and enzymes involved in this bioconversion is still lacking.

Research on carotenoid-related signals in chloroplast stress responses is among this field’s most intriguing and urgent topics. Progress is somewhat hindered by challenges such as the discovery of novel bioactive apocarotenoids and their modifications and the characterization of new biosynthetic and signaling pathways related to the chloroplast stress response in plants. Future research should focus on discovering bioactive molecules (e.g., apocarotenoids) and characterizing their physiological functions. Additionally, elucidating the biosynthetic pathways of apocarotenoids, their compartmentalization, and the molecular mechanisms implicating their regulation in plant chloroplast stress responses must be the focal points for the coming years.

In summary, the stress response markers discussed herein play a pivotal role in plant adaptation to environmental stressors. The analyses of those markers provide insight into plants’ physiological and biochemical pathways to cope with adverse conditions. The detection and quantification of these markers help understand the mechanisms of stress response and inform breeding programs to improve stress resilience in crops.

Combined with other technologies such as NMR, next-generation sequencing, and CRISPR/Cas9 gene editing, MS-based metabolomics strategies are poised to discover new plant bioactive apocarotenoids (including β-CC derivatives and LCDAs) and to investigate the potential biosynthetic and signaling pathways of both novel and known apocarotenoids. High-resolution (HR)-MS/MS is crucial in untargeted metabolomic studies for identifying unknown metabolites, as its ability to measure mass accurately allows for distinguishing different isobaric species while providing structural information through MS/MS analyses. Consequently, HR-MS-based untargeted metabolomics of wild-type plants and their relative mutants, or plants under various stress conditions, can yield valuable insights into promising candidates.

Combined with transcriptomic data, these findings enable the investigation of potential metabolic pathways through high-sensitivity (HS)-MS-based targeted metabolite analysis in wild-type plants and available transgenic lines. While a few pioneering studies have explored these approaches, information regarding the molecular mechanisms remains limited or incomplete. Only a tiny fraction of the described apocarotenoids have been studied in depth, indicating a “virgin” niche for discovering new signaling molecules involved in plant chloroplast stress responses. Uncovering the molecular mechanisms that regulate these compounds to enhance plant stress tolerance will be key to manipulating the content of chloroplast stress-related active apocarotenoids in the future. Integrating transcriptomics, proteomics, and MS-based metabolomics will be instrumental in exploring the signaling pathways that regulate environmental stress responses in plants.

## Data Availability

The original contributions presented in the study are included in the article/supplementary material, Further inquiries can be directed to the corresponding author/s.
